# Evaluation of Hemodialysis Arteriovenous Bruit by Deep Learning

**DOI:** 10.3390/s20174852

**Published:** 2020-08-27

**Authors:** Keisuke Ota, Yousuke Nishiura, Saki Ishihara, Hihoko Adachi, Takehisa Yamamoto, Takayuki Hamano

**Affiliations:** 1 Department of Nephrology, Gamagori City Hospital, Gamagori 443-8501, Japan; 2 Department of Medical Engineering, Gamagori City Hospital, Gamagori 443-8501, Japan; kenkenpa221202@gmail.com (Y.N.); 1214isk@gmail.com (S.I.); toseki@city.gamagori.lg.jp (H.A.); yamamoto-takehisa@city.gamagori.lg.jp (T.Y.); 3 Department of Nephrology, Nagoya City University Graduate School of Medical Sciences, Nagoya 467-8601, Japan; hamatea@med.nagoya-cu.ac.jp

**Keywords:** hemodialysis patient, deep learning, arteriovenous fistula, shunt sound, artificial intelligence, auscultation, convolutional neural network

## Abstract

Physical findings of auscultation cannot be quantified at the arteriovenous fistula examination site during daily dialysis treatment. Consequently, minute changes over time cannot be recorded based only on subjective observations. In this study, we sought to supplement the daily arteriovenous fistula consultation for hemodialysis patients by recording the sounds made by the arteriovenous fistula and evaluating the sounds using deep learning methods to provide an objective index. We sampled arteriovenous fistula auscultation sounds (192 kHz, 24 bits) recorded over 1 min from 20 patients. We also extracted arteriovenous fistula sounds for each heartbeat without environmental sound by using a convolutional neural network (CNN) model, which was made by comparing these sound patterns with 5000 environmental sounds. The extracted single-heartbeat arteriovenous fistula sounds were sent to a spectrogram and scored using a CNN learning model with bidirectional long short-term memory, in which the degree of arteriovenous fistula stenosis was assigned to one of five sound types (i.e., normal, hard, high, intermittent, and whistling). After 100 training epochs, the method exhibited an accuracy rate of 70–93%. According to the receiver operating characteristic (ROC) curve, the area under the ROC curves (AUC) was 0.75–0.92. The analysis of arteriovenous fistula sound using deep learning has the potential to be used as an objective index in daily medical care.

## 1. Introduction

To purify the blood of patients undergoing hemodialysis, a connection from a venous blood vessel to a machine is required. Dialysis, which requires the insertion of two needles to draw blood and two to return blood, is performed three times a week to compensate for lost kidney function. Over several years of continuing dialysis, the blood vessel pierced by the needle gradually narrows [1], a condition called stenosis. The vessel is further narrowed because of the turbulence caused by non-physiological blood flow [2,3,4]; eventually, it may become occluded. Hence, stenosis should be detected, prevented, and treated as early as possible so that occlusion and dialysis delays can be prevented.

The arteriovenous fistula examination is conducted for detection, which involves physical findings by palpation, auscultation, and visual inspection to find the suspected stenosis. If a site exists where stenosis is suspected, the next step is to perform vascular ultrasound or angiography to ensure stenosis detection. After diagnosing stenosis, puncturing should be avoided, as it would further deteriorate the blood vessel from needle puncturing during dialysis. Specifically, puncturing should be avoided in that area to prevent the degree of stenosis from developing further. In addition, a treatment called percutaneous transluminal angioplasty (PTA), which inflates a stenosis site with a balloon catheter and expands it before occlusion, is performed [5,6,7]. To detect stenosis and prevent obstruction, daily consultations called arteriovenous fistula management are essential.

In arteriovenous fistula management, a physical examination using palpation, or palpation and auscultation, is used to check for vibration related to proper blood flow, as well as to determine whether the vessel pulsation is smooth and the pulsation rate is inflated. In auscultation, the presence or absence of constriction is determined by using the pitch difference of sound, discontinuity, spread of sound, and the sound source from other parts (e.g., whistle sound) [8]. In the medical field, this descriptive method is subjective and differs depending on the skill level of the observer and the facility. Novices may only be able to provide a simple description on the presence or absence of constriction sounds. Conversely, skilled persons may record abstract abnormal sounds, such as harmonics and intermittent sounds, in their observation records. As a patient is not assessed by the same skilled observer each day, it is not possible to apply the exact skilled assessment technique daily. Moreover, only subjective results can be communicated to the subsequent examiner. Therefore, the results of auscultation alone do not form a definitive diagnosis of stenosis. Stenosis is diagnosed after the results that have numerical certainty are obtained by performing ultrasound and invasive contrast examination, which require time for preparation and examination. Auscultation is inexpensive, simple, and convenient. However, judgment is delayed if the examiner is not skilled, and the result is abstract. Therefore, auscultation remains only a screening test.

Nevertheless, if auscultation is used as an index that can be output as an objective numerical value rather than as a subjective index, and a diagnosis result with a certainty close to that of an ultrasound examination or a contrast examination is obtained, stenosis diagnosis becomes easy in daily medical care. In addition, if the auscultation technique of a skilled person becomes mechanized, stable output results can be obtained every day, minute changes can be digitally captured and compared with past findings, and the diagnosis accuracy will be improved. The skill of an expert can be reproduced with a machine. Achieving that goal was the primary objective of this study.

There have been reports that stenosis may be estimated during auscultation by using arithmetic processing of the sound frequencies generated by arteriovenous fistulas [9,10,11]. Although it is a mechanical judgment, it is typically only a frequency analysis treble and bass judgment. The arteriovenous fistula sound grasped in the actual medical field is a multidimensional and abstract interpretation. Therefore, the frequency alone is considered not sufficient as a criterion. Conversely, the spectrogram representing the sound source by the frequency density per time interval is assumed to include all the sound elements [12].

**Figure 1:**
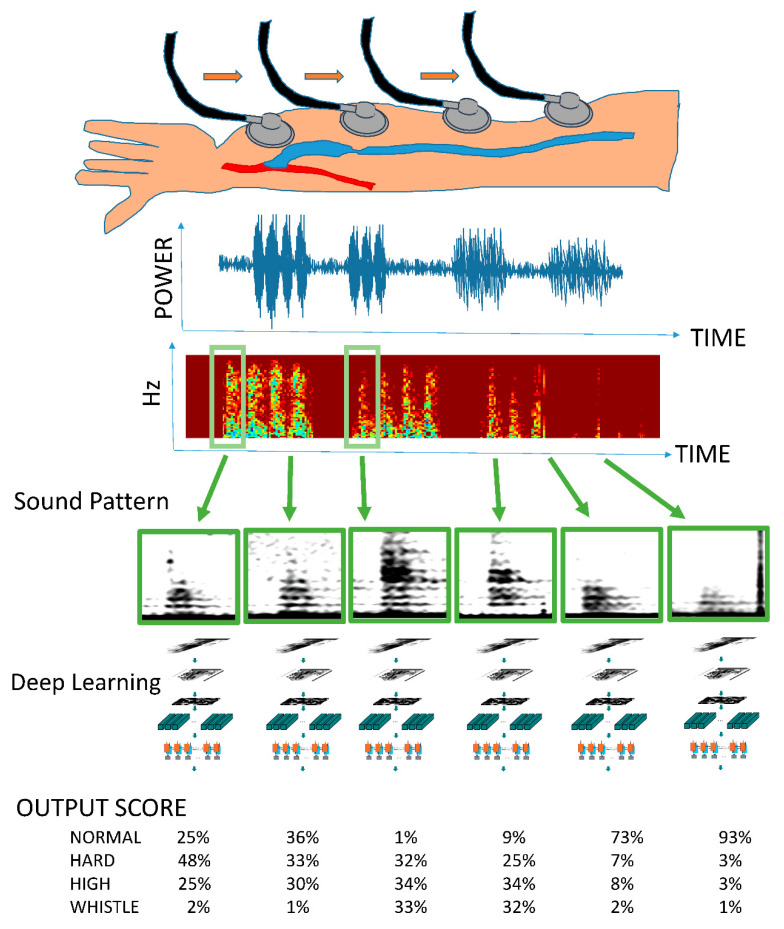
Outline of the arteriovenous fistula sound learning model. A series of arteriovenous fistula sounds are recorded in one medical examination. All continuous tones are converted into spectrograms. A single heartbeat is detected using the mechanism of object detection (R-CNN: regions with convolutional neural networks) similar to that used for face detection in digital cameras and smartphones. The spectrogram of one arteriovenous fistula sound is used as input data. A deep learning model is used for learning, and the importance of the sounds obtained during a general medical examination is expressed by multiclass classification.

To date, objectively digitizing and comparing multidimensional data in familiar environments have been difficult. However, deep learning, which is the basis of image recognition technology and has made remarkable progress in recent years, can be used on personal computers, and, thus, easily comparing multidimensional data is possible. Moreover, it is now feasible to objectively compare spectrograms with audio recordings and images. Accordingly, to evaluate the sound generated by stenosis, we applied a deep learning method using multidimensional data, similar to a spectrogram converted from a arteriovenous fistula sound, and evaluated it as an objective index. The arteriovenous fistula sound learning model is outlined in Figure 1.

## 2. Materials and Methods

The objective comparison of human consultations supplemented with deep learning requires audio monitoring, which we performed using a DR-100MK TASCAM Ⅲ: 24-bit/192 kHz recording device (TEAC Corporation, Montebello, CA, USA).

### 2.1. Participants

The study involved 20 inpatient dialysis patients from a dialysis center (Gamagori Municipal Hospital, Gamagori, Japan). The participants were undergoing treatment in a single facility intended for patients with end-stage renal failure who were undergoing dialysis using arteriovenous fistulas with stable hemodynamic autologous blood vessels. Participating patients were hospitalized for various diseases and were continuing with maintenance dialysis. Arteriovenous fistula sounds were recorded before puncture. Table 1 presents the characteristics of the participants.

All subjects gave their informed consent for inclusion before they participated in the study. The study was conducted in accordance with the Declaration of Helsinki, and the protocol was approved by the Institutional Ethics Committee of Gamagori Municipal Hospital (No. 506-2). Patients on catheter dialysis, patients on dialysis with artificial blood vessels, patients with unstable circulatory dynamics, and other patients deemed by the attending physician to be inappropriate for study participation on medical grounds were excluded.

### 2.2. Methods

#### 2.2.1. Data Preprocessing: Extraction of Single Beat of the Arteriovenous Fistula Sound

The sampled 1-min arteriovenous fistula auscultation sounds also contain variable environmental sounds; therefore, to extract the arteriovenous fistula sound, the value obtained by integrating and averaging the 2–750 Hz region (frequency range characteristic of arteriovenous fistula sound) from each auscultation sound was output [13]. Spline curves were created to reduce the effect of noise, which can cause difficulty in detecting the maximum and minimum values for one beat [14]. To exclude the times during which auscultation was not performed, a convex range falling within a period of 0.5–2 s was estimated to constitute one beat [15].

The sounds determined using a deep learning classifier as being produced by arteriovenous fistulas with a probability exceeding 50% were extracted as the sounds for one arteriovenous fistula beat. Individual arteriovenous fistula sounds recorded for 10,000 beats were classified into one of five categories of sound audible to the human ear (i.e., normal sound, hard sound, high sound, intermittent sound, and whistling). The arteriovenous fistula sounds classified by the human ear had minimal abnormal sounds, including whistles and intermittent sounds. The sound of the whistle sounds like a whistle, which refers to the turbulence in blood flow due to a sharp decrease in the diameter of blood vessels. Intermittent sounds indicate a discontinuity in vascular noise during diastole. It shows a strong obstruction that can cause a complete interruption of blood flow during diastole. High sounds, hard sounds, and normal sounds are often encountered during regular medical examinations. The high sound indicates high-frequency blood vessel noise. It is believed to denote that the diameter of the shunt vessel is thin over long distances. A hard sound is produced by a substantial increase in the amount of vascular noise at the peak, and is considered to indicate arteriovenous fistula vascular resistance physiologically. The collected sound sources were divided into two groups: Test data and training data. We ensured that sources from patients included in the training data were not included in the test data group. Although the training data should have had a large number of samples, the total number of training data was adjusted to be small because the number of abnormal sounds was minimal.

**Figure 2:**
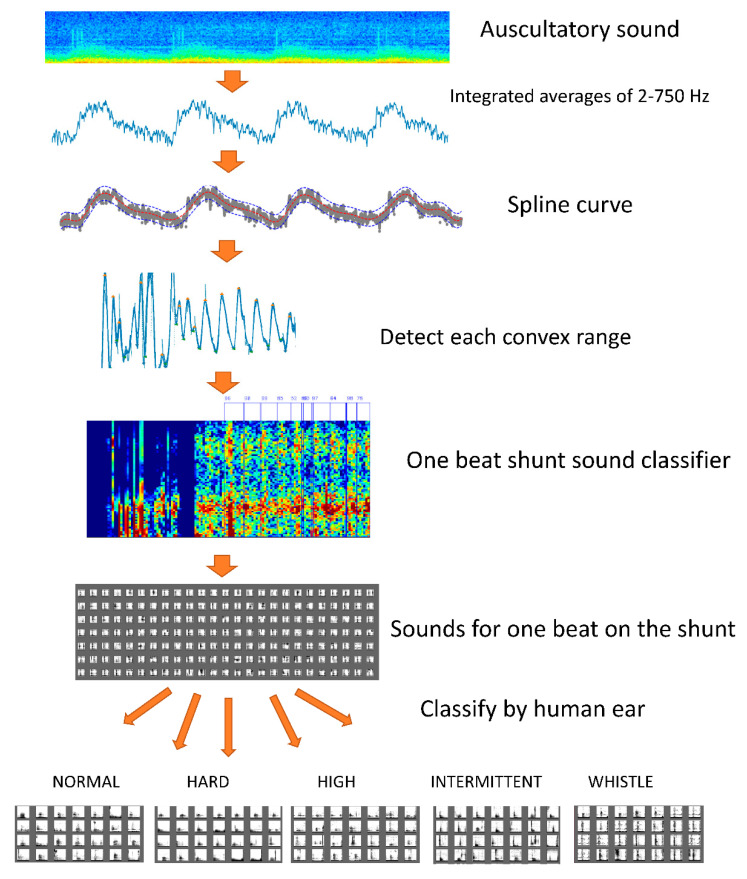
Learning preprocessing. A spline curve was created to eliminate noise from one auscultation sound. We extracted a number of convex curve ranges from the created spline curve with a duration of 0.5–2.0 s. The result was estimated to include an arteriovenous fistula sound equivalent to one heartbeat. The sound of one beat of the arteriovenous fistula was extracted by a deep learning classifier. Arteriovenous fistula sounds of 10,000 beats were classified into one of five types (i.e., normal sound, hard sound, high sound, intermittent sound, and whistling).

In addition, we used class weights that change the weights during learning so that the adverse effects of imbalanced data were minimized. Table 2 presents the contents of the data classified by the human ear. A dataset of 4000 sounds, containing minimal background noise and exhibiting representative characteristics, was used as the learning source. Figure 2 illustrates an outline of the learning preprocessing.

#### 2.2.2. Data Analysis

In the field of deep learning, the Kaggle Competition is an online platform in which statisticians and data analysts from around the world compete to determine who has developed the best models. A competition involving audio tagging took place in 2018. Participants were challenged to develop a machine that can identify common sounds, such as a dog barking, telephone ringing, or guitar being strummed. The Surrey CVSSP DCASE 2018 Task 2 system (an open-source program published on GitHub, MIT licensee) ranked third in the competition and was used in this study for sound classification, as we are familiar with Keras, i.e., the programming language used to develop the program, and the ability of the model to evaluate changes in the time series of sounds [8]. During the actual competition at Kaggle, the sound source provided was 44 kHz; however, this time, we recorded at 192 kHz. The learning effect was expected by capturing fine rumble sounds with a high-resolution spectrogram. According to the product standard, the frequency that the stethoscope can collect is limited by the diaphragm to approximately 700 Hz at the maximum. However, when comparing the auscultation sounds recorded at 192 kHz, there was a wide range of frequencies that changed the sound pattern in synchronization with the beat of the arteriovenous fistula. These included a spiked short keystroke sound up to 3000 Hz, and the arteriovenous fistula sound, which converged to a constant frequency, was 1500 Hz maximum.

Therefore, the frequency axis of the spectrogram used for learning was set to 2000 Hz. The sample size in the Fourier transform was 192 KHz, the window function was 4096, and the hop size was 2048. Feature extraction from the spectrogram was performed by the Mel filter bank. The number of filter banks was increased from the standard 64–1024 to ensure that the effect could be confirmed by a high-resolution conversion, and a comparison was conducted. The collected sound source had a long sound when the heartbeat was slow and a short sound when the heartbeat was fast. Therefore, blanks were filled with blanks for short beats so that the feature amount on the time axis was constant. Gated recurrent unit (GRU) and long short-term memory (LSTM), which use time series data, were used for learning. A small number of features on the input time axis led to a small number of features on the convoluted time axis. The number of features on the time axis before and after the convolution was 400 and 12, respectively. Table 3 shows the learning models used in this system.

## 3. Results

### 3.1. Learning Curve

**Figure 3:**
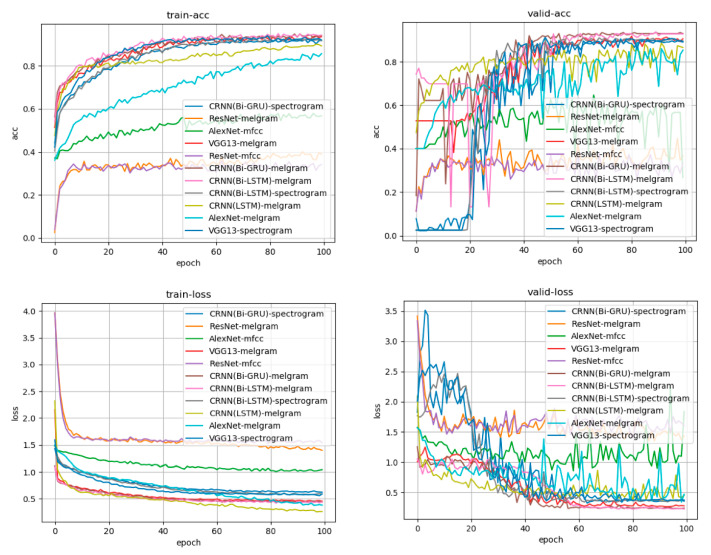
Learning curve for each learning model and each input source. Accuracy is presented in the upper row, whereas the loss is in the lower row. The left side is the transition based on the training data, and the right side is the transition based on the verification data. The horizontal axis indicates the number of times of learning, and accuracy increases as learning progress. Loss represents the difference between the answer of the input data predicted by the model during the learning process (e.g., the degree of firing that is a high tone) and the teacher’s answer to the actual input data (the high tone is the correct answer). It can be observed that the difference between the answer and answer from the learning model obtained during learning has decreased. The CRNN:Bi-GRU model, which had the mel-frequency log spectrogram as input, was the learning model with good accuracy and loss in both training data and verification data.

We compared various learning methods, such as AlexNet, VGG13, a deep residual network (ResNet), and a convolutional recurrent neural network (CRNN), with a bidirectional gated recurrent unit and bidirectional long short-term memory (Bi-GRU, Bi-LSTM). We also compared the mel-frequency cepstrum, mel-frequency log spectrogram, and simple spectrogram with the input signal. Reasonable learning accuracy rates were achieved with the CRNN:Bi-GRU model, which proved capable of learning from sources that included a time series as the data source and the mel-frequency log spectrogram as the input source. Figure 3 shows a graph of the learning process.

### 3.2. Postprocessing

When the five types of sounds (i.e., normal, hard, high, intermittent, and whistling) were individually analyzed, a model suitable for each sound was found. Receiver operating characteristic (ROC) curves were created using the model that best fits each sound. Comparing the area under the ROC curves (AUCs), the model best fitting high and intermittent sound was the GRU with short-term memory; high sounds were best classified using a CNN that could identify rumble sounds. Table 4 lists AUCs classified by each learning model and their corresponding input data features.

**Figure 4:**
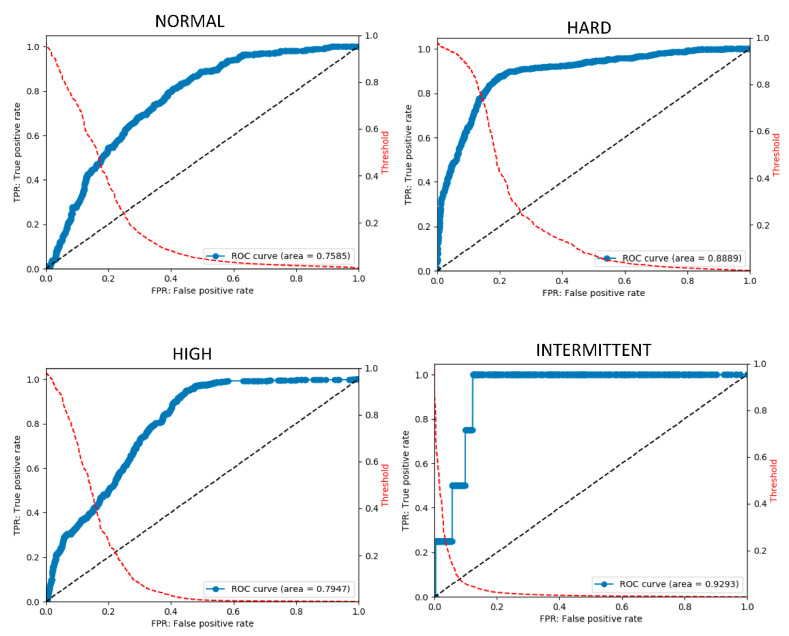

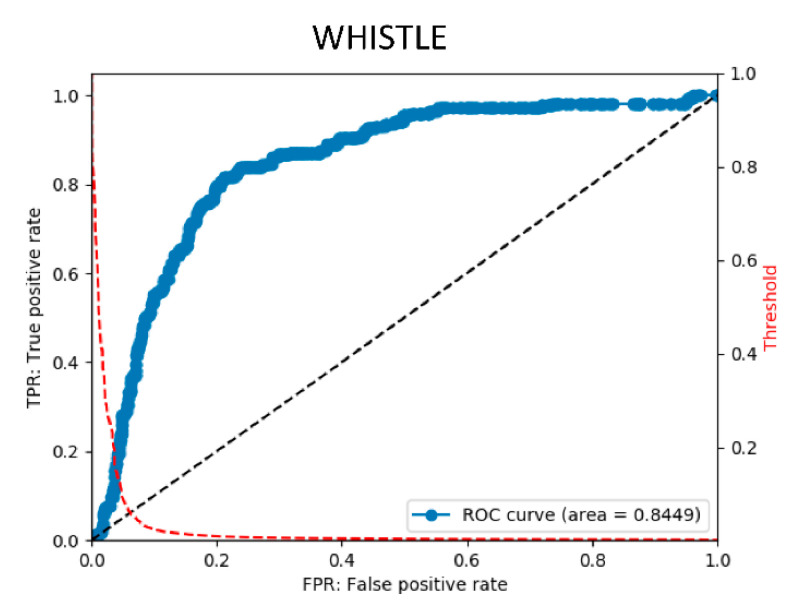
Final ROC curve obtained using the final stage model. The ROC curve is obtained by changing the threshold and plotting the true positive rate (TPR) at each threshold on the vertical axis and the false positive rate (FPR) on the horizontal axis. The ROC curve is on the blue line. The red dotted line shows the curve for obtaining the FPF at each threshold. It can be observed that, by changing the threshold, the threshold can be adjusted to lower FPR to detect rare diseases.

An ensemble method combining judgment outputs was developed using a model advantageous for each sound. The results of the five classifications of unknown data performed by the created classifier are as follows. For each sound type listed above, the accuracy was 75–93%, the sensitivity was 46–86%, and the specificity was 81–93%. According to the ROC curve, the AUC was 0.75–0.92. These results are reasonably reliable for evaluating arteriovenous fistula sounds. Table 5 summarizes the final scores. Figure 4 shows the final ROC curve for each sound type.

### 3.3. Clinical Application

**Figure 5:**
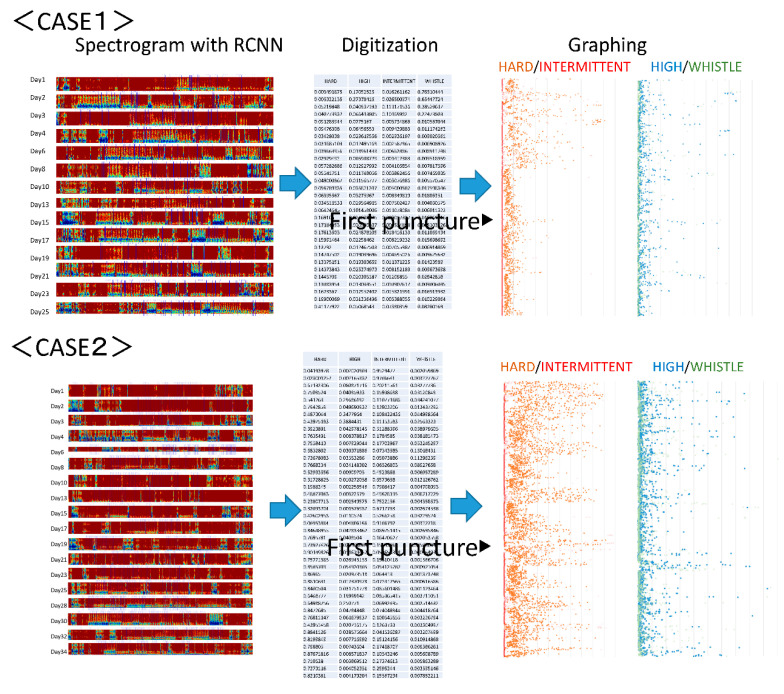
Arteriovenous fistula analysis sound of two cases. The first case is arteriovenous fistula construction resulting from acute renal failure. The puncture began two weeks later. Immediately after creation, the initial sound had a small hardness component due to the influence of a vasospasm. In the second case, an arteriovenous fistula was constructed after a long history of diabetes. The puncture began three weeks later. In both cases, it was observed that the arteriovenous fistula sound was hard at the beginning, even when the arteriovenous fistula sound developed, and that the ratio of random intermittent and harmonic sounds due to the start of puncturing increased.

The analysis of arteriovenous fistula sounds in the two cases that followed the developmental progress starting from arteriovenous fistula creation and continuing for one month is discussed in this section. Figure 5 shows the output results. The first case is that of arteriovenous fistula construction resulting from acute renal failure, with the puncture starting two weeks later. In the second case, the arteriovenous fistula was constructed after a long history of diabetes, and the puncture was initiated after three weeks. The latter artery was a multilayered, highly calcified vessel. The former was affected by vasospasm immediately after arteriovenous fistula creation [16], so the initial sound had a small component of hardness. Thereafter, in both cases, it was observed that a hard sound was conspicuous at an early stage, even when the arteriovenous fistula sound first developed. Moreover, the ratio of random intermittent and harmonic sounds increased after the start of puncturing. This may have indicated an increased risk of arteriovenous fistula stenosis after puncture [17].

## 4. Discussion

Complicated arithmetic processing and statistical analyses can now be easily implemented owing to the improvements in computer processing ability. Although examples have been applied clinically in various medical fields, clear test protocols, such as diagnostic imaging, blood sampling, genetic testing, electroencephalograms, and electrocardiograms, are still required [18,19]. Conversely, in daily practice, doctors tend to place less emphasis on physical examinations that provide only subjective records and place greater emphasis on blood sampling or imaging tests that provide objective results [20]. Conventional physical examination findings and other physical results are subjective, making it difficult for examiners to vary examination techniques and compare findings with past results to make assessments simply and quickly. Mechanization of medical examinations will soon be an important aid for guiding such inspections. However, input devices that are capable of determining tactile pressure, heat sensation, and response to pain are still evolving, hindering their integration with electronic devices. These findings rely on human hands to convert and digitize the data. However, auscultation, as performed in a classic medical examination, can be integrated with digitization using current medical technology.

There have been reports on the use of deep learning for digitizing visual examination results in dermatology, as well as in analyzing heart sounds for the estimation of heart disease [21,22,23]. However, this approach requires that only useful heart sounds should be extracted for a consultation record, and the preprocessing required for a trained classifier also hinders use in clinical settings. In this study, we used a region-detection classifier that detects a single beat from the preprocessing stage for the deep learning of arteriovenous fistula sounds. The required arteriovenous fistula beat sound was detected from the stored long-term auscultation sound without prior knowledge of the record, and the sound could be connected to the stenosis judgment classifier as carefully selected judgment data material. Deep learning using a carefully selected one-beat arteriovenous fistula sound has facilitated the objective digitization of the subjective evaluation performed by the main examiner.

In addition, by converting simple scientific findings into objective numerical values, it becomes possible to obtain frequent objective medical data. In the area of arteriovenous fistula examination, frequent observation may allow stenosis caused by a puncture to be found in the early stages of arteriovenous fistula development. Multidimensional data that were originally difficult to compare and evaluate are not limited to storage as images but could be digitized for medical examination evaluation. Thus, this practice may be applied to other medical fields if an input device is developed.

**Figure 6:**
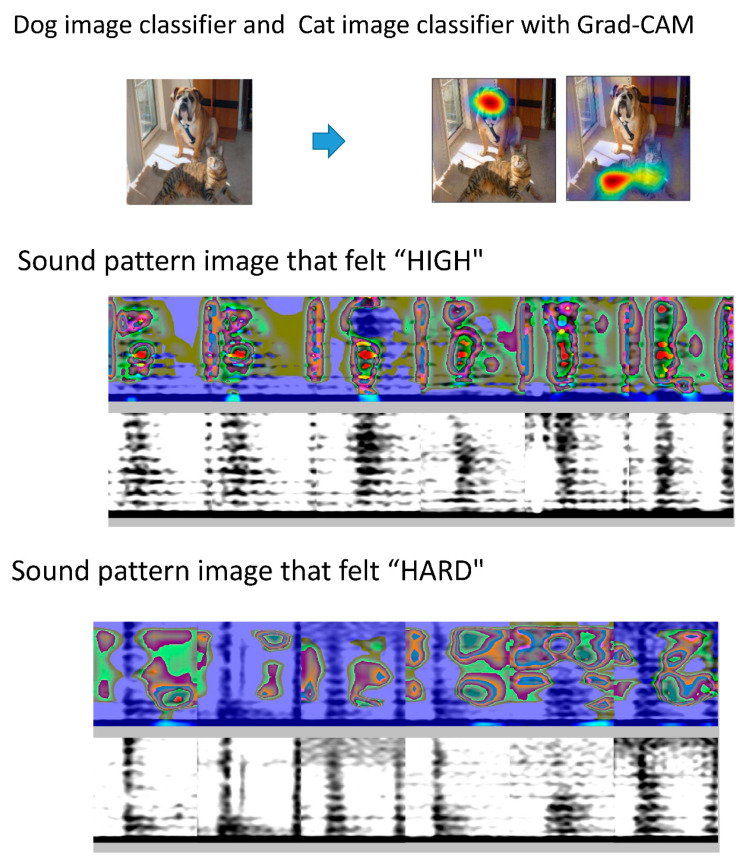
Grad-CAM for visualization of characteristic sites. With Grad-CAM, if the image is judged to be a dog using a dog image classifier, the image is colored. The same applies to a cat image classifier. When the characteristic portion of the sound pattern image that felt “high” is visualized, the 250–750 Hz region in the systole is emphasized in the heat map. In the case of a sound pattern image that felt “hard,” the silent region in the diastolic area was emphasized.

However, classifications based on black-box artificial intelligence methods remain unclear. An example of this is Grad-CAM, which identifies the feature points that CNN selects, focuses deep learning on the sound pattern image [24], and highlights these features in a heat map. In a different application, using a “dog” image classifier, color is applied to the characteristic portion of test images determined to be those of a dog. Figure 6 shows a concrete image output by Grad-CAM. In our case, when the characteristic portion of the sound pattern image that felt “high” was visualized, the 250–750 Hz region in the systole was emphasized in the heat map. When the characteristic part of the sound pattern image that felt “hard” was visualized, the silent region in the diastolic area was emphasized. It can be observed that the rumble region in the high-frequency region is used as an index, which is consistent with a previous report on the features in the spectrogram of stenosis sounds [25]. In the intermittent periods, the classifier focuses on the silence, and intermittent sound can be indexed as an element that allows a person to recognize a hard sound.

One limitation of this study is that the number of cases used was small; thus, the number of abnormal sounds collected was also small. During the learning phase, learning was performed by weighting the class of abnormal sounds; however, the extent of the performance handicap was unknown. In clinical practice, the disease frequency is low, approximately once every several thousand times. Regardless of how effective the classifier is, the number of judgment results will be false positive or false negative. Either can be prioritized by changing the cutoff value of the classifier; nonetheless, the effect is a tradeoff. As a countermeasure, in the medical field, a population with a higher pre-test probability in other items can be extracted and applied to the judgment machine. In this arteriovenous fistula sound classification approach, a large classifier that combines the classical statistical method based on physical sound features [26] and a learning model that detects abnormal sounds by unsupervised learning with this learning model is considered to be an effective classification method [27,28,29].

The system used in this case was a multi-class classification system that distinguishes the sound from everyday sounds. The arteriovenous fistula sound may have harmonic, intermittent, and whistle sounds that simultaneously overlap. Thus, we should have changed the system to multi-label classification. Moreover, the diagnostic accuracy did not increase although it was a normal sound with a high proportion. The concept of normal sound is an excluded item that is not abnormal, and the feature amount is difficult to extract. The arteriovenous fistula sound should be a regression problem that numerically outputs the degree of stenosis, not a classification problem. The numerical value output by the system based on this classification problem is not proportional to the degree of stenosis. For example, the number output in the item of harmonics is a percentage representing the possibility of being the average harmonics collected this time. If the value is high, it does not mean that the constriction is a strong harmonic. When the average harmonics collected by the training data are compared with the other groups, the ratio of the average harmonics is shown. As the teacher data determines the presence of harmonics, realistic recursive numerical values are difficult to output.

In future examinations of the risk of stenosis, arteriovenous fistula auscultation should not be studied in only one time period. However, the hard sound before stenosis, the high and whistle sounds that signify stenosis, and the normal sound after stenosis is resolved should all be studied [30]. It will likely prove necessary to construct a model for arteriovenous fistula stenosis diagnosis that uses a higher-dimensional time-series learning model, instead of the single-beat model. In addition, the accuracy rate for the existing model was high. However, it decreased when presented with new data, indicating inadequate generalization. Additional data collection periods may be required until a small number of abnormal sounds have sufficient data to facilitate classification. Finally, evidence confirming that this method can realize the ultimate goal of assisting with diagnoses is insufficient; consequently, comparisons with existing ultrasound resistance indices and a comparison before and after PTA remain necessary in clinical practice [31].

## 5. Conclusions

Arteriovenous fistula auscultation can be substituted using deep learning methods with high accuracy. However, arteriovenous fistula consultations require careful evaluation in conjunction with visual inspection, palpation, and other types of information. As the training data used for deep learning are also influenced by the audio of various devices and environmental characteristics of the facility, assessing and improving the reliability, efficacy, and cut-off scores of these methods require multi-center studies with larger populations.

## Figures and Tables

**Table 1:** Sociodemographic and clinical characteristics of the 20 participants. Participants included patients between the ages of 49 and 90 with various diseases.

Characteristic		Value
Age (median (IQR), years)		73 (63–79)
Gender (n)	Male	13
	Female	7
Dialysis duration (median (IQR), years)		0.01 (0–19)
Cause of end-stage renal disease		
Diabetes		11
Glomerulonephritis		5
Others		4
Type of arteriovenous fistula		
Radiocephalic AVF at front arm		17
Radiocephalic AVF at mid forearm		2
Brachiocephalic AVF at elbow		1

Abbreviations: AVF, arteriovenous fistula; IQR, interquartile range.

**Table 2:** Content of the data classified by the human ear and input data used for learning and training. Training and test data were categorized to avoid similar input data from the same patient.

	Training	Test	Total
Normal	394	485	879
Hard	578	901	1479
High	670	563	1233
Intermittent	91	4	95
Whistle	91	217	308
Total	1824	2170	3994

**Table 3:** Various learning models used for the input source. The basic structure of all learning models comprises a neuron model called VGG13. First, the input data (Log Mel spectrogram) to VGG13 is convolved with 64 filters of 3 × 3 size, and the same convolution is repeated. Next, a rectified linear unit (ReLU) is used as the activation function, and Batch normalization (BN) is performed on the obtained data. Subsequently, the information is compressed using a 2 × 2 size filter called max pooling. Then, the same operation is repeated by gradually increasing the number of filters so that fine features can be captured effectively. Finally, all the obtained data are combined, the activation function (softmax) is used to stimulate 5 neurons, and the output value represents classification into 5 classes. When using convolutional recurrent neural network (CRNN), we use data convoluted by VGG13 as the input data before fully combining them. The output results using the bidirectional gated recurrent units (Bi-GRU) or bidirectional long short-term memory (Bi-LSTM) of the neuron model, which can evaluate the time axis, are connected to the fully connected layer and classified into five classes.

VGG13	CRNN (Bi-GRU)	CRNN (Bi-LSTM)
Log mel spectrogram	Log mel spectrogram	Log mel spectrogram
3 × 3, 64, BN, ReLU	3 × 3, 64, BN, ReLU	3 × 3, 64, BN, ReLU
3 × 3, 64, BN, ReLU	3 × 3, 64, BN, ReLU	3 × 3, 64, BN, ReLU
2 × 2 Max Pooling	2 × 2 Max Pooling	2 × 2 Max Pooling
3 × 3128, BN, ReLU	3 × 3128, BN, ReLU	3 × 3128, BN, ReLU
3 × 3128, BN, ReLU	3 × 3128, BN, ReLU	3 × 3128, BN, ReLU
2 × 2 Max Pooling	2 × 2 Max Pooling	2 × 2 Max Pooling
3 × 3256, BN, ReLU	3 × 3256, BN, ReLU	3 × 3256, BN, ReLU
3 × 3256, BN, ReLU	3 × 3256, BN, ReLU	3 × 3256, BN, ReLU
2 × 2 Max Pooling	2 × 2 Max Pooling	2 × 2 Max Pooling
3 × 3512, BN, ReLU	3 × 3512, BN, ReLU	3 × 3512, BN, ReLU
3 × 3512, BN, ReLU	3 × 3512, BN, ReLU	3 × 3512, BN, ReLU
2 × 2 Max Pooling	2 × 2 Max Pooling	2 × 2 Max Pooling
3 × 3512, BN, ReLU	3 × 3512, BN, ReLU	3 × 3512, BN, ReLU
3 × 3512, BN, ReLU	3 × 3512, BN, ReLU	3 × 3512, BN, ReLU
	Bi-GRU, 512, ReLU	Bi-LSTM, 512, ReLU
Global average pooling		
Softmax (5 classes)		

**Table 4:** Comparison of the areas under the receiver operating characteristic (ROC) curves (AUCs). A list of AUCs classified by each learning model, each input feature, and each sound is presented. For each sound, the item with the maximum AUC is highlighted. Hard has the best score in the GRU model using 1024 features. High is the model using 128 features, intermittent is the GRU model using 1024 features, and whistle is the CNN and GRU model using 64–256 features.

Feature	64			128			256			512			1024		
	CNN	GRU	LSTM	CNN	GRU	LSTM	CNN	GRU	LSTM	CNN	GRU	LSTM	CNN	GRU	LSTM
**Normal**	0.58	0.59	0.60	0.70	0.72	0.64	0.69	0.73	0.66	0.69	0.72	0.66	0.70	0.72	0.65
**Hard**	0.70	0.70	0.68	0.81	0.81	0.69	0.84	0.87	0.78	0.83	0.90	0.85	0.83	**0.91**	0.73
**High**	0.77	0.77	0.76	**0.80**	0.77	0.78	**0.80**	**0.80**	**0.80**	0.78	**0.80**	**0.80**	0.79	0.76	0.77
**Intermittent**	0.83	0.88	0.77	0.78	0.85	0.82	0.83	0.78	0.77	0.87	0.87	0.82	0.84	**0.94**	0.92
**Whistle**	**0.89**	0.85	0.85	**0.89**	**0.89**	0.86	**0.89**	**0.89**	**0.89**	0.87	0.87	0.87	0.88	0.88	0.86

**Table 5:** Final score. The final score is obtained by ensembling a plurality of models in which the best score is recorded for each sound using a technique called stacking and using the final stage model. The correct answer rate for the test data was unknown 72–93% (average 82%), and the AUC was 0.75–0.92% (average 0.83%).

	NORMAL	HARD	HIGH	INTERMITTENT	WHISTLE	MEAN
accuracy	0.753	0.833	0.729	0.935	0.855	0.821
precision	0.45	0.766	0.478	0.014	0.36	0.414
recall	0.468	0.862	0.478	0.5	0.581	0.578
specificity	0.836	0.812	0.817	0.936	0.885	0.857
f1	0.459	0.811	0.478	0.028	0.444	0.444
AUC	0.759	0.889	0.795	0.929	0.845	0.843
